# Exploring salutogenic mechanisms of an outdoor experiential learning programme on youth care farms in the Netherlands: untapped potential?

**DOI:** 10.1080/02673843.2014.896267

**Published:** 2014-04-14

**Authors:** Ester Schreuder, Mandy Rijnders, Lenneke Vaandrager, Jan Hassink, Marie-José Enders-Slegers, Lynne Kennedy

**Affiliations:** a Department of Psychology, Division of Clinical and Health Psychology, Utrecht University, Utrecht, The Netherlands; b Department of Social Sciences, Health and Society Group, Wageningen University, Wageningen, The Netherlands; c Plant Sciences Group, Wageningen University, Wageningen, The Netherlands; d Faculty of Psychology and Educational Sciences, Open University of The Netherlands (OUNL), Heerlen, The Netherlands; e Department of Clinical Sciences and Nutrition, University of Chester, Chester, UK

**Keywords:** outdoor experiential learning, Salutogenesis, care farms, behavioural problems

## Abstract

This study explored how (learning) experiences offered through outdoor experiential programmes, particularly the youth care farm approach, may (or may not) enhance young peoples’ ability to recognise and then utilise available resources for personal growth, protection and health promotion. A total of 11 youngsters were asked to look back on their half-year stay on a care farm in the Netherlands, by using semi-structured interviews to elicit their experiences from a salutogenic perspective. Analysis revealed that several resources (and the interaction of these resources) on the youth care farm worked well for the youngsters; contributed to their personal development and to their sense of coherence: the feeling that the world is or can be meaningful, comprehensible and manageable, associated with positive outcome in endeavours linked to improving health and well-being. In general, the attitude of the farmer, working with animals, the informal atmosphere and being temporarily cut-off from the former environment were elements most positively highlighted by the youngsters. The farm environment was mentioned as calming, however, as structuring as well. The strength of the programme as an experiential learning opportunity appears to be the diversity and richness of resources (and stressors!) available to the participants. This creates various opportunities for learning: making sense, interpreting and giving meaning to resources and stressors. Further research into the impact of this kind of programmes, compared to more ‘traditional’ programmes, especially on the ability of youngsters to use resources to finish school, find employment and develop better relationships with their parents is recommended.

## Introduction

The social, emotional and mental well-being of young people is an important determinant of both their own health, personal development and social relationships, and of those around them. In the Netherlands today, one out of five youngsters and their families are directly and indirectly affected by inadequate or ‘dysfunctional’ social, emotional and mental development and the so-called ‘behavioural problems’ (Schrijvers & Schoemaker, 2008).

Young people experiencing serious behavioural problems are typically referred into youth care facilities or interventions, ranging from day care to weekend stay and family homes, offering individual or group counselling. For some, however, the extent or nature of their problem is such that prevailing youth care programmes are ineffective (Hassink, 2010). For these ‘troubled youth’, alternative forms of youth care such as outdoor experiential programmes, including youth care farms, may provide a valuable, yet untapped, resource in the promotion of health and well-being for young people and their families (Ploeg van der & Scholte, 2003; Russell & Phillips-Miller, 2002).

Evidently, the unique and effective component of outdoor experiential programmes such as wilderness challenge programmes (Wilson & Lipsey, 2000), wilderness therapy (Harper, Russell, Cooley, & Cupples, 2007), outdoor behavioural healthcare treatment (Russell, 2003) and adventure programming (Hans, 2000) is the emphasis on physically challenging activities in the outdoor environment, such as backpacking or rock climbing, combined with treatment grounded in the field of experiential learning (Gillis & Gass, 1993; Wilson & Lipsey, 2000). Researchers have shown experiential learning to yield benefits in terms of improved mental and social health (Harper et al., 2007; Russell & Phillips-Miller, 2002), reduced antisocial and delinquent behaviour and increased locus of control and self-esteem (Hans, 2000; West & Crompton, 2001; Wilson & Lipsey, 2000). This concurs with reports on the importance of physical effort and a therapeutic component (Russell & Phillips-Miller, 2002; Wilson & Lipsey, 2000), the green environment (Hassink, Elings, Zweekhorst, van den Nieuwenhuizen, & Smit, 2010), task accomplishment, restructuring of the therapeutic relationship, development of a therapeutic social group and the use of self-reflection (Russell & Phillips-Miller, 2002). Of importance is also the focus on the positive assets of young people rather than problematic behaviour. For example, Kirschman, Roberts, Shadlow, and Pelley (2010) concluded that interventions that enhanced feelings of optimism and hope were health-enhancing for adolescents and their families. Of interest to care farms is the importance of a trusting relationship developed between adolescents and mentors, and the development or enhancement of social support (Bettmann & Tucker, 2011; Greeson & Bowen, 2008; Williams, 2000). As Becker (2010) highlights, the neutral (non-institutional) environment and unorthodox approach to intervention helps eliminate barriers associated with traditional therapeutic settings like stigma, intimidation and mistrust resulting from differential power relationships.

Youth care farms are an innovative example of the outdoor experiential programmes for youth with emotional and behavioural problems and are now being considered by youth care as a potential alternative to traditional programmes in this area of work (Hassink et al., 2010). Care farming is a growing movement that combines agricultural production with health, social and educational services. Care farms offer day care, supported workplaces and/or residential places for different clients with a variety of disabilities (Hassink et al., 2010; Hassink, Zwartbol, Agricola, Elings, & Thissen, 2007). Youth care farms are locations where youngsters and farmers’ families live and work together. In the Netherlands, some 100 youth care farms provide services to more than 200 youngsters annually (Hassink, Meyer de, Sman van der, & Veerman, 2011).

Youth care farms offer a context that is new for experiential programmes. The experiential learning programme on youth care farms in the current study is a ‘living and working programme’ developed by a youth care organisation in the Netherlands. They work with youngsters with severe behavioural problems between the ages of 16 and 23. It starts with an individual survival-training course followed by six months of living and working on the farm. This programme is unique and differs from most common outdoor experiential programmes: the length of the programme is longer than most, the approach is client-centred and the roles of the farmer and his family as non-professional caregivers are carefully defined to offer social support. Initial research on these unique characteristics show promising results in terms of potential benefits (Hassink et al., 2011). Knowledge concerning the interplay of factors that contribute to these outcomes is lacking. The same applies to comparable outdoor experiential programmes in youth care settings: although the experiences with outdoor experiential programmes are generally promising (Ploeg van der & Scholte, 2003), understanding the link between positive outcomes and the programme itself is unclear (Russell, 2006) and warrants investigation.

## Theoretical framework

Understanding of how individuals cope with adverse situations or circumstances is explained by many theorists. The medical sociologist Aaron Antonovsky devoted his work to understanding the link between resources and antecedents that ‘create health’ (Salutogenesis), as opposed to the prevailing and hegemonic focus amongst health and social care professionals on what causes disease (pathogenesis). Antonovsky (1987) introduced Salutogenesis to identify and explain health-enhancing and protective factors essential for an active and productive life. He attempted to address the salutogenic question ‘why some people, regardless of major stressful situations, are able to live an active and productive life, while others do not’. The answer was formulated in terms of the sense of coherence (SOC) and general resistance resources (GRRs) (Lindström & Eriksson, 2010). The SOC refers to a general life orientation (Antonovsky, 1987). Three separate concepts contribute to the SOC: (1) comprehensibility – a belief that things happen in a predictable fashion and a sense that you can understand events in your life and reasonably predict what will happen in the future; (2) manageability – a belief that the resources are available and that things are manageable and within your control; and (3) meaningfulness – a belief that there is good reason or purpose to care about what happens (Antonovsky, 1987; Eriksson & Lindström, 2005). People have access to a variety of the so-called ‘general resistance resources’ (GRRs) which one can use to strengthen his SOC. These resources that are at someone's disposal can be internal or external, material or immaterial. Examples are self-confidence, knowledge, social environment or money (Lindström & Eriksson, 2010). Salutogenesis studies the dynamics between people and environment and how health develops from this interaction – or not. It aims to identify patterns and mechanisms underlying health processes, rather than factors bound to people. Society can be rich in resources, the outcome depends on how people make sense, interpret and give meaning to these resources in relation to health (Bouwman & Swan, in press).

### Applying Salutogenesis to experiential learning

Experiential learning seems to offer resources which can be significant to strengthen the SOC. The objective of this study is to explore how (learning) experiences offered through outdoor experiential programmes, particularly the youth care farm approach, may (or may not) enhance young peoples’ ability to recognise and then utilise available resources for personal growth, protection and health promotion. In order to get insight into the underlying mechanisms and resources, the current study aims to describe the characteristics of this programme, and how these are experienced by the youngsters.

**Box 1: Details of the ‘living and working programme’ (hereafter referred to as the programme)**

**Participants**

The ‘living and working programme’ is developed for youngsters aged between 16 and 23 with severe social and mental health problems, varying from externalising (acting out, e.g. aggression) to more internalising problems (inward, e.g. anxiety and mood disorders, social withdrawal). The young people concerned face problems in the following domains: (1) family (they cannot stay at home due to aggression and running away), (2) school or work (they do not attend school or have no job) and (3) friends and free time (they do not spend their free time in a constructive way). Their days are filled with hanging around, abuse of alcohol and drugs and criminal activities. Contraindications for participating in the programme are severe psychiatric or addiction problems, risk for severe aggression, insufficient abilities for reflection and lack of motivation. General objectives of the programme are to develop more positive perspectives in the domains of ‘functioning’ (e.g. school, work or family life), while developing a workable relationship between youth and parents.

**Introduction: survival (two days)**

Youngsters start the programme with an individual survival trip in the Ardennes (Belgium) for two days, accompanied by a youth care worker. They must complete exercises, such as making a bivouac for the night, alongside many physical challenges. Important aspects of this survival are (successful) experiences and taking responsibility: a first step in reflecting on their own behaviour.

**Living and working on the farm (six months)**

Period 1 – the focus is on adjusting to living and working on the farm. Objectives are learning to listen to the farmer and maintaining their own living unit. Contact with family and friends is restricted.
Period 2 – youngsters compare their actual situation with past experiences. Weekly telephone contact with parents is allowed.
Period 3 – focus is on reflection of changes in behaviour over this time. More contact with parents is allocated.
Period 4 – focus on making plans for the future. Youngsters stay with parents during two weekends.

During this whole farm period, a counsellor from the youth care organisation visits the youngster twice a week. Progress, problems and assignments are discussed; the farmer is involved to reflect on the state of affairs in the youngster's functioning on the farm. Personal reflection on their development is an important element of the programme. Youngsters keep a daily diary and are encouraged to reflect on actual and previous situations. They also receive training in aggression regulation or social skills. Parents are required to participate in classes to enhance parenting skills.

**Aftercare programme (six months)**

Both the individual living and working on the farm and the training for youngsters and parents are the basis for the aftercare programme. In most cases, youngsters return to live with their parents. A counsellor of the youth care organisation visits the youngster on a weekly basis. Learning goals related to improved functioning in school, work and free time are discussed and agreed between youngster, parents and counsellor.

## Methods

### Study design

This paper reports on the findings of a qualitative inquiry, using semi-structured interviews to elicit youngsters’ experiences of a ‘living and working programme’ in the Netherlands from a salutogenic perspective. This study builds on earlier research (Hassink et al., 2011) conducted in the same programme.

### Recruitment

Out of 102 youngsters, 74 graduated from the programme operating at care farms in association with youth care organisation (Hassink et al., 2011). A cohort of youngsters who completed the programme (2009–2010) was invited to participate in the present study (*N* = 25). They were contacted by letter and telephone, via the youth care organisation, to explain the aims of the research and recruit to the study. A total of 11 youngsters agreed to participate, 9 men and 2 women, (age range 17–22) (Table 1). Two youngsters refused participation due to negative experiences in the programme; two were not able to participate because of personal circumstances; remaining rejections concerned practical reasons (e.g. work).

**Table 1.** Description of the sample (*n* = 11): gender and mean age.

	Number (*n*)	Age (Mean)
Female	2	18.5
Male	9	19.6
Total	11	19.36

### Research methods

Semi-structured interviews were used as the primary method for data collection. An interview guide was developed to explore the experiences involved in behavioural changes of youngsters participating in the programme, with particular interest to resources of the programme at the farm and how youngsters make sense, interpret and give meaning to these resources. Areas covered included youngsters’ personal situation (e.g. family background and education/work), details of the programme at the farm (e.g. farm characteristics, motivation and expectations, goals, mentoring) and general reflections on the programme (e.g. experiences, significance, what specific farm-related issues were important to individual participants). In the interviews a funnel method was used (Bowling & Ebrahim, 2005): at the start, open-ended, rather general questions were asked and during the interview questions became more specific and detailed. In this way the interviewee was guided by his or her own experiences, uncontrolled by the interviewer, allowing dynamics within the interview to inform the direction of conversation. Emphasis was placed on empathetic and inductive data collection (Zwieten & Willems, 2004).

### Procedures

Interviews were held at participant's home address or at a preferred alternative by prior arrangement. Informed consent was obtained from all participants prior to the interview. Permission to record the conversation was also requested. Interviews typically lasted one hour and all participants received remuneration. Interviews were transcribed verbatim by the researchers and personal information was anonymised. The data were put into qualitative data management software Nvivo 9.2 (Bazeley & Jackson, 2013).

### Data analysis

Data analysis was conducted using interpretative phenomenological analysis methodology (Boeije, 2010), which was chosen for its suitability to explore a research area extensively in which the perceptions and experiences of the participant (not the researcher's) are prioritised (Smith & Osborn, 2003). Transcripts were double coded and compared, and data were clustered into related themes according to the principles of thematic analysis. Themes were created inductively and deductively (reference to SOC framework); overlapping and new or emerging themes discussed by the research team. Saturation was reached when no new themes were found and no relevant new labels could be attributed to the transcripts. Since two participants experienced completely different situations in their programme (e.g. the placement was not based in the Netherlands), these were removed from the analysis. Nine interviews were included in the dataset.

## Findings

Participants’ accounts of the care farm experience were collected. Particular attention to the salutogenic mechanisms of their care farm experience is reported here with reference to Salutogenesis and the three components of comprehensibility, manageability and meaningfulness.

### Comprehensibility

Most youngsters participating in the programme led a turbulent life, characterised by problems and contact with several youth care organisations and institutions. Although the programme offers possibilities for change, this requires youngsters to understand and accept the overall aims of the programme. As this youngster discovered, only when he understood the link between the work and his ‘recovery’ did the relevance make sense:

I had never worked a day in my life and then this farmer I don't know suddenly says like ‘yes you must’ you know, and he gives me one of those things. He says ‘scoop the droppings’. In the beginning I thought like, what are you talking about you know. I don't know you, I won't scoop those droppings for you for free and all that. That's why at first I said, the first month was like … not difficult, but I just didn't do the things that were asked of me, I didn't understand why. Why do I have to do that? … Sometimes when you talk about it and all, it's just gonna be okay. Then he explains to me why or what, how, you see? Okay. I will do it, blah, blah. You surrender, that's it. (4)

Experiencing hard work and taking responsibility for animals enabled some to discover the satisfaction of doing a job well:

Sure. In the beginning, yes, I never worked a day in my life so yeah that was quite difficult, but after a while you get used to it and get yourself out of bed and you just won't be late anymore. It's just life. It is their daily life, and when you are involved and there is no distraction then you get used to it, simply by myself, that's not difficult. (4)

The role of comprehensibility also emerged around discussions of social skills or aggression-regulation training. Many did not appreciate why they should participate or how they would benefit. Only when the youngster comprehended the importance of the training to his overall ‘recovery’ was he fully motivated and engaged.

The temporary interruption or reduction of contact with friends and family made youngsters see the influence some people had on them. Individual placement on the farm seems to play an important role in a growing awareness of one's personal functioning and behavioural patterns. The youth care farm in this way offered a starting point for reflection and behavioural changes:

Yes, watching TV a bit. That's all there is to do anyway. Watching a bit of TV or listening to the radio. Yeah, I mostly just watched TV … It was really boring, you had no friends, you just see nobody no more. Yeah, that was difficult. You then start missing your parents and all, and you start thinking about how many mistakes you've made and all, and the mistakes made by your parents. Yes, all that is difficult … you know, during Christmas, Boxing Day, when you are used to be with family and then you're there staring at a Christmas tree on your own. (8)

This way of working (with assignments) did not seem to suit every youngster:

Well, I didn't find it very useful for me, all those assignments…. I always find it a bit weird and then you have to make these assignments of which you should learn something or so…. What should I learn from keeping my diary or uh, I don't exactly know what kind of assignment I had to make, but, I think every youngster experienced that as an obligation … And that they don't really make progress by doing that … and when I was with the farmer himself, we were just talking. That helped me a lot more than filling in some questions or making a few assignments. (5)

### Manageability

A relation between manageability and the programme on the youth care farm can be seen in the way the youngsters spoke about the activities on the farm. At the start of the programme, these tasks and the daily routine on the farm were completely new for the youngsters. Adjustment was required, which was a big challenge for most of them. However, as the youngsters settled into their role as an employee on the farm, their defiance and incomprehension decreased. They learned that what was expected of them was not unreasonable, and that they were able to cope with it. This showed the youngsters how much they could handle, as long as the tasks are manageable. This understanding gave the youngsters an experience of success and resulted in a growing feeling of control over their own lives:

Uh, those working hours were okay like that, from 7 am to 7 pm, sometimes, sometimes it was too long, because sometimes you had to work longer, half past 7, eight o'clock. But then, I was only 15 years old … that is heavy! To do that … and once until 10 pm, when all those chickens had to be slaughtered, you worked until 10 pm, I was really exhausted. I really didn't feel like it anymore. And then still the farmer made me work [further]. Pshaw … But, everything turned out well … (8)

On the other hand, a lack of this feeling of manageability could also lead to a lack of motivation to continue the programme. For example, one of the youngsters did not experience a good relationship with the farmer. Another did not feel supported by the peasant family. The activities the farmer asked of these youngsters were harder to execute when they felt overloaded:

The only thing that counted was work, and furthermore I couldn't share anything with them you know. (…) I just needed some compassion, and I couldn't find that in the family, because they simply had no sympathy with me. (2)

The dynamic character of enhancing the SOC becomes apparent in this case: the youngster made use of other sources that played an important role in his track, like the contact with the mentor and support from family and friends. Another example of manageability became apparent in the regulation of behaviour. Some of the youngsters linked their own behaviour to the way the farmer and his family dealt with conflicts or workload. One of the youngsters reported that he learned not to run away in conflict situations, but to communicate about problems and talk things out. Another youngster mentioned that he learned ‘to stay calm, for that farmer did so’. Discussing events with the mentor was also important for most:

Yeah, uh, it wasn't always easy to talk about that sort of things, but, in the end it was actually very helpful to talk about those things…. Yeah, the way you react yet changes so to say…. Then you respond this way, and then you respond very different so to say. And then what is the better way to respond so to say…. but I think that eventually if there is continually insisted on and all, that eventually, you will respond in another way…. Yes I know now about myself that it's like that…. Because they just insisted on certain things very much, and yeah, I just notice now that I respond very differently on it than three years ago. (1)

In this way, some youngsters have learned new and lasting communication skills. Others, however, still experience conflicts in their family environment and showed less ability to translate the various skills or resources of the project into behavioural changes in their daily lives. Most of the youngsters did not receive aftercare from the youth care organisation. In some cases, this had to do with the transition to another form of youth care. As the experiences of this youngster shows, ongoing support from his mentor, after the stay on the farm, was important in helping the transition and changes being more manageable:

She came at my home every two weeks to have a talk and all…. and with my parents as well, about if there had been any conflicts or so, then we spoke about how we solved them and all. and that's why it's better now…. Yes, half a year at the farm, half a year here at home. So she was my coach for a year…. Yeah, then it was finished. Over and out, everyone was gone. Hop! (8)

### Meaningfulness

This component of the SOC relates to how youngsters perceive the various elements of the programme. Important here is the personal experiences of the different elements and how individuals intended to draw upon these resources in future. On the whole, the youngsters named comparable sources as important or meaningful. For example, all youngsters reported it as meaningful that the farmer put his trust in them by giving them responsibility in their tasks on the farm. This trust confirmed their value as an employee on the farm. They experienced success in their work:

I just went with the farmer every time. And he just gave me a few tasks and then I just did that on my own, the whole day, when he did something else. He just left me alone in the stables and all, and then he went somewhere else … Yeah, I don't know, I liked it that he gave me that trust … that was indeed something new. When you come to somebody and that guy knows what you all did than he doesn't just easily trust you anymore. And if he then acts like that, yeah that is quite something … yeah trust, that somewhere he actually trusts you. (3)

Mutual respect seems to play an important role in this matter:

He didn't regard you as a criminal, you know. (9)

Working with animals became especially meaningful for some youngsters. Some youngsters emphasised that animals are ‘relaxed’ and non-judgemental, in contrast to comments received from people on the farm. Experiencing the responsibility of looking after animals was also linked to learning goals:

The farmer, he constantly reminded me that I didn't, I couldn't give up, and he also said, yeah, you can't say like, I put my stuff down and I quit. Who will feed those animals then? … If you don't do that, the animals will die. And that's just something where you, well, you just start to realise, that walking away is simply not an option, is just no possibility. (2)

In some cases, the activities offered the youngster new future prospects. An example concerns a youngster who began to appreciate working with horses and now aspires to work in this field:

Working with horses made my future you know. Because I am doing that education now, and eh, working with horses really is the thing I like the most out of everything I have done so far. (6)

Another youngster showed his family that he was able to work hard, and subsequently considered to register for an education. The support and encouragement that youngsters experienced from their mentor, family and friends proved to be important for their functioning on the farm. One of the youngsters, for instance, mentioned how conversations with his mentor encouraged him to carry on with the programme and to complete the six months:

My mentor, who coached me during the whole programme … she really supported me a lot as well, and she also told me many times like come on, you can do it … She came by two times a week. And then we first had a joint conversation with the farmer and his wife, and then things were discussed that went well, that went less well, what I had to work on, and after that I had an individual conversation with her alone for a while, about how I experienced it myself, because she just wanted to know my personal opinion as well. (7)

The same goes for parents who let their children know (by letter) how proud they were, which motivated the youngster to live up to this trust and therefore stick to the programme:

A nice feeling that people still think about you and send you mail. (2)

Lack of the habitual contact with friends made clear who were really supportive.

You see, back then I really thought they were my real friends. And, not one of them wrote me a letter or something like that while I was there, so that made me think like, yeah, real friends, my ass. (1)

It's nice that, despite all that time, and often you then notice that there are a lot of friends that break contact, that your friends stay loyal to you despite everything. (7)

Another example of meaningfulness regards the individual placement on the farm. Being cut-off from their home environment could on the one hand be experienced as difficult, while on the other hand proved to be a source of reflection.

In half a year time you just get so confronted with yourself, you have so much time to think about what you've been doing, and that actually it's almost impossible not to come to the realisation that what you're doing is just not the right way, and at some point you just start thinking like, I just want to chase a new direction in my life. (2)

Youngsters came to realise in what way their friends influenced them in their lives. Some of the youngsters seemed to translate this insight to their functioning after the programme on the farm, and were able to differentiate more in their friendships. Others, however, ended up on the street again and relapsed into earlier problem behaviour. One of the youngsters described how a relapse in drug use after the programme startled and disappointed him so much that he immediately stopped and never did it again.

## Conclusion and discussion

Lack of methodological description and theoretical basis is a serious critique on experiential learning programmes (Cason & Gillis, 1994; Russell, 2003). The goal of this study was therefore to explore mechanisms and resources underlying health-promoting processes of an experiential learning programme on a youth care farm. Results are considered in the light of Salutogenesis and its concepts of the SOC and GRRs (Antonovsky, 1987; Eriksson, 2007; Lindström & Eriksson, 2010).

Qualitative analysis revealed that several resources (and the interaction of these resources) of the experiential learning programme on the youth care farm worked well for the youngsters; contributed to their personal development and to their SOC: the feeling that the world is or can be meaningful, comprehensible and manageable. In general, the attitude of the farmer, working with animals, the informal atmosphere and being temporarily cut-off from the former environment were elements most positively highlighted by the youngsters. The farm environment was mentioned as calming, however, as structuring as well.

Experiences with the formal assignments that youngsters were required to undertake as part of their training, commissioned by the youth care workers, were predominantly negative. Most of the youngsters disliked ‘having’ to complete a diary, while others had already participated in similar programmes or courses on developing social skills or aggression regulation and consequently were less motivated to revisit such topics. Restrictions associated with the stay on the farm, such as limited contact with family and friends but ‘forced’ contact with the farmer and his family, were sometimes evaluated negatively. On the other hand, these negative feelings had an unanticipated outcome in motivating youngsters to make extra effort to demonstrate their worth and contribution. This ‘stress’ also stimulated reflective learning processes.

In terms of the salutogenic resources associated with the experiential programme, those resources that evoked (1) comprehensibility (during the programme) – a belief that things happen in a predictable fashion and a sense that you can understand events in your life and reasonably predict what will happen in the future – were: (i) the activities on the farm, (ii) the interaction with the farmer, (iii) the assignments given by the mentor and (iv) the individual placement on the farm with associated time for reflection. Resources that evoked (2) manageability (during the programme) – a belief that the resources are available and that those things are manageable and within control – were: (i) the tasks on the farm with the guidance of the farmer and (ii) the examples set by the farmer and the mentor concerning communication skills. Resources that evoked (3) meaningfulness (during the programme) – a belief that there is a good reason or purpose to care about what happens – included: (i) the trust and responsibility given by the farmer, (ii) the interaction with and care for the animals, (iii) the experienced support and encouragement from mentor and from family and friends and (iv) being cut off from family and friends.

The strength of the programme as an experiential learning opportunity appears to be the diversity and richness of resources (and stressors!) available to the participants. This creates various opportunities for learning: making sense, interpreting and giving meaning to resources and stressors. The health-enhancing and protective resources, identified in the interviews, did not work for every adolescent in the same way or to the same extent. Some of them mention the relationship with the farmer or the counsellor of the youth care organisation, while for others the bond with farm animals is important. When one of the potential resources, like a good relationship with the farmer, does not develop, it can be compensated by another resource.

### Other research

These findings are consistent with previous research which typified elements of the programme such as the farmer as an employer, the farmers’ family, supervision by a youth care worker, the activities on the farm and the characteristics of the farm on which a youngster is placed as promising characteristics (Hassink et al., 2011). The importance of trusting relationships and attention to self-reflection is apparent from some of the literature (Bettmann & Tucker, 2011; Greeson & Bowen, 2008; Williams, 2000). The farmer and the mentor have different roles in the programme, which seem to complement each other well. In line with previous studies, the farmer and farmer's family as non-health-professionals were (by several youngsters) appreciated as good listeners, reliable, supporting and respectful (Piat, Wohl, & Duruisseau, 2006; Walter & Petr, 2006). Interaction with farm animals was important to some of the youngsters in this programme. This concurs with previous studies highlighting the support for people suffering from loneliness and isolation and mistrust (Ewing, MacDonald, Taylor, & Bowers, 2007; Mallon, 1994). The study also confirms the importance of useful work: giving structure to the day, a social identity, status and support (Boardman, Grove, Perkins, & Sheperd, 2003).

### Limitations

In terms of the value of the research, several aspects are worth consideration. First, the participants in this study were invited by the youth care organisation, which may introduce bias in terms of outcomes as the organisation invited youngsters who they believed were more likely to participate and consequently may have had a more positive view of the programme compared to youngsters not invited. Second, even though all the youngsters had behavioural problems, the underlying problems and/or psychopathology were diverse. Finally, the study was retrospective and carried out approximately 12 months after the programme finished. An interview held directly after finishing the programme would probably provide us with more or other information. Memory recall bias and also a change in perception over time tend to occur when interviewees are asked about experiences retrospectively. Attempts were made by the researcher to clarify meaning in respondents’ feedback and avoid recall bias, where possible; this limitation may need consideration when interpreting our findings.

### Future research

Youth care farms appear to provide a range of resources that contribute to the personal and social development of young people with severe social and mental health problems, providing opportunities to develop in a way that life becomes more comprehensible, manageable and meaningful. Continued research in this field is recommended to further explore and understand potential benefits. The matching between youth care farm and the youngster is made quite intuitively. Based on research findings, a better fit between farm and farmer characteristics and adolescents with their specific problems could be developed, for example. One of the strengths of the programme is the non-institutional setting where the farmer and the counsellor of the youth care organisation have different roles; the farmer guides the adolescent in the work and the counsellor in the achievement of objectives. As such, it is an interesting example of combining professional and informal/lay care. It would be interesting to further investigate how both types of guidance can be integrated in an optimal way. Currently, youth care farms are used as a last alternative for youngsters with severe problems. Based on the results of this study, care farms could also play an important role at an earlier stage in child development to prevent problem behaviour or promote healthy development before adolescence, when behaviour is more difficult to influence.

Further research into the impact of this kind of programmes, compared to more ‘traditional’ programmes, especially on the ability of youngsters to use resources to finish school, get a job and develop a good relationship with their parents, is recommended. Furthermore, the sustainability of the effects of youth care farms should be researched since it is not clear to what extent positive benefits and outcomes are maintained after completing the programme.

It can be concluded that the setting of this programme offers multiple resources, which are experienced and used differently by the youngsters. As such, youth care farm programmes can be a valuable and successful alternative to more traditional therapy forms. This is inherent to the concept: the youth care farm as being a rich learning environment for (different types of) young people. The findings show that youngsters are able to make sense of the world around them. This awareness may possibly play an important role in sustainable behavioural changes.
